# Ambulance nurses' experiences of deciding a patient does not require ambulance care

**DOI:** 10.1002/nop2.255

**Published:** 2019-03-19

**Authors:** Tess Backman, Päivi Juuso, Ronja Borg, Åsa Engström

**Affiliations:** ^1^ Ambulance Care Mora Sweden; ^2^ Division of Nursing, Department of Health Science Luleå University of Technology Luleå Sweden; ^3^ Västerås Hospital Region of Västmanland Västerås Sweden

**Keywords:** ambulance care, clinical decision‐making, patient safety, qualitative research, refusal of care

## Abstract

**Aim:**

To describe ambulance nurses' experience of deciding a patient does not require ambulance care.

**Design:**

An inductive, empirical study with a qualitative approach.

**Methods:**

Data collection was conducted through semi‐structured interviews, and collected data were analysed with qualitative manifest content analysis. Data were collected during the spring 2017, and eight ambulance nurses participated.

**Results:**

The findings are presented in one main category, which is “Not very ill but a difficult decision” with totally three subcategories. The ambulance nurse's experience of making the assessment when the patient has no need for ambulance care is like walking the balance of slack line. This means that the assessment can be both easy and very difficult but something that definitely requires experience, knowledge and dedication.

**What does this paper contribute to the wider global clinical community**

- The ambulance nurses' perception of the assessment is not always consistent with the patient's and close relatives' perception of the patient's needs.
- The ambulance nurse sometimes feel insufficient despite his/her intentions of doing well.
- The ambulance nurses experience of safety with the colleagues is important as well as to be able to assist an equal colleague in difficult assessments.
- Guidelines for assessing patient care need to be developed, but even more importantly, it is to develop the cooperation with other healthcare providers.

## 1 INTRODUCTION

Nurses working in ambulance care setting form their initial image of the patient in the assessment based on their experiences and intentions (Holmberg & Fagerberg, 2010). They make an assessment of the patient's situation and background, and examinations of vital parameters such as measurement of breathing, blood pressure and observations such as the patient's skin (Widgren & Jourak, 2011). According to Widgren and Jourak (2011), the majority of Sweden's ambulance operations use the Rapid Emergency Triage and Treatment System trauma system. The triage is carried out in two steps, and a medical evaluation of the patient's vital functions and the patient's main symptom, which consists of different codes and is called Emergency Symptoms and Signs (ESS). The highest level of the two determines the overall triage category. The ESS codes are red, orange, yellow, green and blue. Patients who are classified as red need immediate treatment while patients classified as blue are non‐urgent and are not admitted to hospital (Barfod, Danker, Forberg, & Lauritzen, 2010).

There are several different structured concepts to follow in the pre‐hospital assessment. Advanced Medical Life Support (AMLS) is a concept that helps the nurse to identify life‐threatening states early in the assessment and effectively manages a wide range of medical situations (National Association of Emergency Medical Technicians, 2014). Internationally as well as nationally, the number of non‐urgent patients using ambulance care increases (Barrientos & Holmberg, 2018; Rosén, Persson, Rantala, & Behm, 2018). More than 15% of the patients remain at home after assessment of ambulance staff. They get recommendations for self‐care or to seek care at different level of care than hospitals (Hjälte, Suserud, Herlitz, & Karlberg, 2007a; Norberg, Wireklint Sundström, Christensson, Nyström, & Herlitz, 2015). Carret, Fassa, and Kawachi (2007) show that resources in the form of alternative care levels are not utilized to a sufficient extent or is not available. According to Hjälte, Suserud, Herlitz, and Karlberg (2007b) one third of the patients transported to emergency department are estimated by the ambulance staff to have been able to ride a taxi or their own car. In recent years, the number of ambulance transports in Sweden has increased by more than 25% (Krey, 2014). Research show that some patients did not really need ambulance transport (Challen & Walter, 2010; Hjälte et al., 2007a), while Tohira et al. (2016) ask if it is appropriate for patients to be discharged by scene by ambulance staff? Höglund, Schröder, Möller, Andersson‐Hagiwara, and Ohlsson‐Nevo (2019) described these decisions complex and trying. Vloet et al. (2018) found that patients not in need for ambulance are younger, often lives in rural areas and initial reasons for care relates to mental disorders. In summary, in Sweden and in other countries, the ambulance nurses need to be prepared for emergency care of critical ill patients, but also for them not in need of ambulance care. The aim of the study was therefore to describe ambulance nurses' experience of deciding a patient does not require ambulance care.

## 2 METHODS

### 2.1 Design

The present study is an empirical study with a qualitative approach.

### 2.2 Context

The study's participants are employed at an ambulance station in northern central Sweden. During 2016, the number of ambulance missions in the county (part of Sweden) where the ambulance station belongs to was a total of 37,097 missions. Of these, 9,550 missions were issued from the selected ambulance station. During 2016, nearly 20% of patients at the current ambulance station were assessed in place that the permit did not require ambulance transport. According to Péclard (2016), the most common is that these assessments are performed at a residential address, and the second most common places are work, street/road or other public place.

### 2.3 Participants

The inclusion criteria were that participants would have more than 1 year's work experience as ambulance nurse and experience of making judgments when the patient's condition did not require ambulance care. Ten participants were asked to participate in interviews, of which eight left their consent for participation, five were females and three men. The participants were between 26–61 years, had 5–29 years' experience in ambulance care and had worked as ambulance nurses for 1.5–16 years. They had all a specialist education in ambulance and pre‐hospital care at advanced level.

### 2.4 Ethics

Permission to conduct the study was given by the head of the ambulance station. The Local University Ethical Review Board approved the study. The head of the ambulance station contacted ten ambulance nurses and informed them about the study. These 10 people received a letter containing information about the study and that the participation was voluntary, confidential and that they could withdraw from the study any time without explanation. The letter also included an invitation to participate; eight chose to do so, and they gave written permission for the researchers to contact them for an interview by signing a consent form. All data have been processed and kept confidential between the authors.

### 2.5 Data collection and procedure

The participants were chosen due to their experience of patients assessed not being in need of ambulance. The interviews were made at the agreed time and were digitally recorded. The interviews took place during the spring of 2017 and lasted between 20–45 min. The participants were asked to describe their experiences of patients who they assessed not being in need of ambulance care.

### 2.6 Analysis

A qualitative content analysis with inductive approach was performed. The content analysis was conducted in a number of steps where the manifest content of the text was preserved (Graneheim & Lundman, 2004). The interviews were transcribed by the authors who then read the whole text and discussed the meaningful units that responded to the aim, and these were extracted. These meaning units were cut down without losing their meaning, known as condensation, and they were then given a code, which briefly described the core of the content. Codes with similar content were then sorted into four subcategories. The text as a whole in these subcategories could be summarized under one main category. The authors had all previous experiences of working as ambulance nurses, all had a master degree in nursing and one is senior lecturer and one professor in nursing. The pre‐understanding of the authors were useful for asking the “right” questions, but we were also aware of the risk of making to fast judgements of the data from our previous experience. Therefore, we analysed the data systematically and discussed each step.

## 3 FINDINGS

The findings are presented in one main category: Not very ill but a difficult decision. The category was created from three subcategories presented below (Table 1).

**Table 1:** Overview of the categories (*N* = 2) and subcategories (*N* = 4)

Category	Subcategory
Not severe ill but a difficult decision	A wish to do well
	Feeling the fear of missing something important
	Feeling calm and feeling safe with the colleague

### 3.1 Not very ill but a difficult decision

#### 3.1.1 A wish to do well

It was difficult for participants to postpone the assessment when the patient had no need for ambulance care despite the patient was claiming it. Participants described that sometimes the situation required that the decision should be changed according to the patient's wishes.

It's a bit like going to the balance of a slack line in a way for the patient to feel sick or to have a problem so that it needs help and … so we get there and do not think it may be necessary … (8)

In situations where the patient according to their assessment was not in need of ambulance care but the relatives meant that the patient needed it, was described as difficult situations to solve. Sometimes, the situation was further complicated when the patient did not consider needing to have ambulance care.

Relatives may be a little stubborn … but we have taken on all the parameters, we have assessed the patient to be able to stay at home and the patient also want to stay but relatives say the patient should enter … (2)

Participants could feel questioned in spite of good intentions to explain to patients and relatives why there was no need for ambulance care. The patient's involvement was important in the decision. After a situation where the assessment was questioned by patient and their relatives, participants reflected on whether something could have been done differently.

You can think about it if you might be able to do something different or express yourself in another way next time? If it had been different then? (5)

The participants told about situations where the patient was in need of care from relatives, and the situation in the home became unsustainable when the relatives were in need of rest. The patient did not really have a need of ambulance care. How the situation was resolved then felt ethically complicated. Participants explained that when they saved an old person from the waiting time at the emergency room and instead arranged alternative care contacts, it seemed like a good deed.

I felt that I might should have driven this patient [to emergency department] so the partner could rest, but at the same time I knew that this patient will take a bed that someone who really needed it medically could get, so it´s a society problem as I feel emotionally touched by, you're at a crossroad. (1)

Participants thought it was important to be clear to the patient that the situation was not easy to solve, but the goal was always to make the patient feel safe.

Solving things that do not belong to my duties really, but still trying to solve the problem in a way … or solve the situation so that it will be good for the patient … one will be very happy to help someone in a way that it will be very good … then you will be very happy and like a little raised so to speak. (8)

#### 3.1.2 Feeling the fear of missing something important

The participants told they experienced fear to miss something in the assessment that could be almost impossible to detect. Despite this, they must have confidence in their own ability to make the assessment. The amount of assessments of patients who had no need for ambulance care contributed to personal development that reduced the sense of uncertainty.

If you feel the least insecure … the least hesitation, the patient wants to go in, and we may think that he or she does not have to go with us, and then we will take lightbulb or other transport in course. (6)

Participants wanted better collaboration with other levels of care in order to make it easier to contact the other caregivers and patients' relatives. One wish was to be able to provide a written information sheet and the telephone number that the patient could apply. To be able to read in the patient's journal before or during the patient meeting would facilitate understanding the patient's care history. The participants felt that some patients were more difficult to assess, for instance, worried elderly patients with several diseases with diffuse symptoms. It could also be challenging when language barriers arised between the ambulance nurses and the patients. In the situations where relatives interpreted, it was difficult to know how exactly the translation was.

The hardest thing is when there are language bans when you cannot really understand how to explain to the patient or understand what is really happening … It is by far the most difficult one. (7)

#### 3.1.3 Feeling calm and feeling safe with the colleague

The experience of security was based on experiences of meeting different patients with different disease states. Participants said that experience was something that evolved over several years based on assessments of many patients.

When you do it many times, you will be more confident in the decisions as well. (8)

Participants described how they at first glance, with the so‐called “clinical look,” made a first assessment. They described that “the feeling of power” and the ability to read between the lines were important for the assessment; it is not possible to replace guidelines or flow charts with the “clinical look.”

Maybe it is not that I am just about how the patient is saying or what she or he is saying, but you also have a special feeling and you have met similar patient categories many times and built on an experience. (1)

The treatment guidelines and compliance with the AMLS concept created security and were a support to fall back on in the assessment, even in non‐urgent patients. Participants could experience the patient's trust in the assessment and did not feel like having to meet a physician. They described other examples that created security for them in the assessment: the specialist education for the ambulance nurse as well as exchange of experience with colleagues. Another example mentioned was interpreting services to avoid language delays.

The easiest patient group to assess were young people without deviating symptoms. Participants perceived that it was often the case that the patient was apparently completely unaffected and had relatives who would have been able to request the patient to the appropriate level of care. Only when the participants felt safe in their assessment and the patient was experienced as calm, they left the patient left at site. Participants pointed out that it felt important to clearly inform the patient about the thoughts about the assessment and, if necessary, to alarm again. If the decision after the assessment felt good at the site—the participants felt satisfied.

When I feel completely confident in the assessment, I feel quite excited to yes … well‐being as well as I do … (6)

The assessment felt safer when also the colleague was a registered nurse, instead of nurse assistant, as it facilitated the reasoning about the assessment. Participants described an experience of security in having the colleague as a support and that, as a colleague, it could be a support if there was doubt about the patient's need for ambulance care.

You take control of what you need to do and then you make a decision together with your colleague. (7)

## 4 DISCUSSION

The patient could choose to stay on the site when the assessment showed that there was no need for ambulance care. Porter et al. (2007) show that the patient's right to decide for him/herself is an important part of the decision to leave or bring the patient to hospital, indicating the importance of the patient being able to participate in the decision. In this study, it was sometimes difficult to resist the patient's will. Challen and Walter (2010) argue that patients sometimes require care and ambulance transport despite the fact that the ambulance staff assesses them otherwise. Sandman and Bremer 2016) describe that an ethical problem arises when the patient's will and the staff's judgment conflict with each other. There may be difficulties for ambulance staff in today's judicial community to persuade the patient to remain in place due to that health care is not considered the ambulance transport is necessary (Porter et al., 2007). This may mean that the patient's participation in the assessment reduces the risk of patient and ambulance staff becoming two opposites.

In this study, the participants felt emotionally affected by the patient's situation and felt that they had to help the patient in some way, even though they did not belong to their duties. In the field of ambulance nursing, the primary responsibility of the nurse is to care for patients who need ambulance care (Holmberg & Fagerberg, 2010; Sandman & Nordmark, 2006). Andersson Hagiwara, Suserud, Jonsson, and Henricson (2013) describe that there are times when ambulance staff have to deal with situations that do not belong to their actual duties. Ambulance staff can sometimes meet patients with other needs, such as social needs (Holmberg & Fagerberg, 2010). Nurses describe that they worry about situations where they feel insufficient and cannot help the patient (Jonsson & Segesten, 2004; Svensson & Fridlund, 2008). An ethical dilemma arises after the assessment when the patient has no need for ambulance care and the nurse fails to get in touch with the appropriate care level for example psychiatric care. Since the ambulance is alerted to the location, the nurse experiences a responsibility for the patient (Holmberg & Fagerberg, 2010), indicating a great willingness to do good by helping people. The patient is moving to emergency room whenever the situation is not possible solving. This is because the emergency room is open 24 hr, even though it is not the best solution for the patient, instead the patient might would have felt better if he or she could have stayed at home with support (Holmberg & Fagerberg, 2010). In order to cope with this kind of situation, a great ability to be humble, creative and flexible is required (Ahl et al., 2005; Andersson Hagiwara, Suserud et al., 2013).

The result showed that there was a great willingness to make both patient and close relative pleased. Barrientos and Holmberg (2018) describe that the condition of some patients does not allow ambulance care. Nurses try to find a different solution for the patient than to postpone emergency care in order to make the best possible for the patient. A central task for the ambulance staff is to safeguard the patient's best, which is not always evident when there are several different care options (Sandman & Nordmark, 2006). There is a possibility that the patient's wishes regarding care and transport do not match the principles of ambulance care. A decision that follows the treatment guidelines can be ethical error, according to Gunnarsson and Warrén Stomberg (2009). The result showed that the participants reflected their actions in situations that made an impact on them emotionally. Gunnarsson and Warrén Stomberg (2009) argue that experience is created by reflecting on their own mistakes and is a way to find alternative solutions. By recognizing situations and developing action strategies for similar situations, new knowledge can be acquired, which confirm the importance of reflection on own actions. This study found that the colleague was of importance to the ambulance nurse's experience of the assessment. Svensson and Fridlund (2008) state that knowledge of the colleague's knowledge increases safety. Wireklint Sundström and Dahlberg (2011) show that the assessment of the patient is done jointly with the colleague to facilitate care decision and treatment. Gunnarsson and Warrén Stomberg (2009) describe the role of colleagues in the assessment, where communication, cooperation, security and knowledge of colleagues' experience and judgment prove to be important. Nurses in the ambulance care can feel worried when there is a lack of trust in the colleague (Svensson & Fridlund, 2008). Ambulance staff are looking for colleagues whom they feel confident in order to talk about their feelings (Ahl et al., 2005; Jonsson & Segesten, 2004; Svensson & Fridlund, 2008). Ambulance staff are constantly exposed to new situations where assessments and decisions have to be made; hence, there is an understanding of the collegial importance both practical and in terms of confidence (Ebben et al., 2017).

The experience of making the assessment when the patient had no need for ambulance care was described as a difficult decision. In ambulance care, the assessment of patient needs is sometimes very difficult (Tärnqvist et al., 2017). The biggest challenge for healthcare professionals is to assess patients with diffuse or misleading symptoms (National Association of Emergency Medical Technicians, 2014). According to Porter et al. (2007), sometimes the ambulance staff hesitate over the patient's need for care and then considers whether the patient can stay on the spot. This study showed that participants felt a fear and insecurity to miss something in the assessment. According to Norberg et al. (2015) when information is insufficient to create a comprehensive picture of the patient, the nurse must collect more information. In case of doubtful assessments, the patient is transported to hospitals because the nurse experiences fear of losing his/her identification (Porter et al., 2007; Snooks et al., 2005). When neither the ambulance staff nor the patient has a clear picture of the need for care, the patient may leave the responsibility to the ambulance staff (Porter et al., 2007). Snooks et al. (2005) suggest that it is easier to leave the patient in place after assessing whether the patient has existing contact with primary health care.

Patients who were found to be hard to assess in the results of this study were older people. Vicente, Castren, Sjöstrand, and Wireklint Sundström (2013) interviewed elderly patients who sought emergency services for a certain period. Hospitals are associated with survival and security, a place where expertise and necessary care are gathered. The participants in this study described that when the language advocacy prevailed, the assessment was complicated. McCarthy, Cassidy, Graham, and Tuohy (2013) found that language barriers could limit conversations between patients and healthcare professionals. Hultsjö and Hjelm (2005) describe that the body language is of different importance to different cultures and complicates the assessment of the severity of the state. When relatives interpret the conversation in the assessment, it is difficult for healthcare professionals to know how exactly the translation is (Hultsjö & Hjelm, 2005; McCarthy et al., 2013). Some patient groups were perceived as more difficult to understand, which previous studies reinforce mainly communication. The findings of this study showed that experience is considered the basis for safety in the assessment. According to Porter et al. (2007), knowledge and skills are considered important, but above all experience has a major impact in the assessment of medical conditions. Experience leads to an understanding of different conditions, which shows that both theoretical knowledge and experience are needed to optimize the assessment. Breeman, Poublon, Verhofstad, and Lieshout (2018) conclude that ambulance nurses safely can examine the patient, initiate treatment when required and make decisions about which patients do not need immediate ambulance transportation. Snooks et al. (2005) describe that the ambulance staff's decision not to bring patients is based on experience and intuition as well as the attitude of colleagues and patients. Ambulance staff considered that after many years in the profession, they had sufficient experience and knowledge to make sure that the patient had no need for ambulance care. Another study describes that ambulance staff must take control of the situation, provide peace and security to get an idea of the patient's needs (Wireklint Sundström & Dahlberg, 2011). Bremer, Dahlberg, and Sandman (2012) show that through a professional approach, ambulance staff to the patient can convey peace and security. A calm and safe care creates a calm atmosphere that benefits the situation and the assessment. Booker, Shaw, and Purdy (2015) suggest strategies to assist patients and bystanders in mitigating their perceptions of risk and state that we should be sensitive to the idea that patients and relatives might not know what type of help they need when they contact emergency care services. Rantala, Forsberg, and Ekwall (2018) highlight that within ambulance care, also and maybe especially, when it is non‐emergency, the care should be person‐centred and always taking patients seriously.

### 4.1 Methodological considerations

More than 1 year's work experience as a specialist nurse was considered appropriate as inclusion criteria, when the authors considered it important that the participants had landed in their new role (Benner, 1993). It takes a number of years to develop the ability to reflect on ones assessments rather than focus on current practices like new within the profession.

## 5 CONCLUSION

The ambulance nurses' perception of the assessment is not always consistent with the patient's and close relatives' perception of the patient's needs. In some situations, the ambulance nurse feel insufficient despite his/her intentions of doing well. From the perspective of the ambulance nurse, there is a need for improved cooperation between different levels of care to optimize patient care. This benefits patients to get the right level of care, which increases patient safety and quality of care. The ambulance nurse's experience of making the assessment when the patient has no need for ambulance care is like walking the balance of slack line. This means that the assessment definitely requires experience, knowledge and dedication.

## DISCLOSURE

The authors declare no conflicts of interest.

## AUTHOR CONTRIBUTIONS

ÅE, TB, RB, PJ designed the study. TB and RB collected the data. ÅE, TB, RB, PJ analysed the data. ÅE, PJ, TB, RB prepared the manuscript. All authors approved the final version for submission.
